# Microbe Profile: *Saccharomyces eubayanus*, the missing link to lager beer yeasts

**DOI:** 10.1099/mic.0.000677

**Published:** 2018-09-03

**Authors:** José Paulo Sampaio

**Affiliations:** UCIBIO-REQUIMTE, Departamento de Ciências da Vida, Faculdade de Ciências e Tecnologia, Universidade Nova de Lisboa, 2829-516 Caparica, Portugal

**Keywords:** yeast, lager beer, microbial genomics, evolutionary ecology

## Abstract

*Saccharomyces eubayanus* was described less than 10 years ago and its discovery settled the long-lasting debate on the origins of the cold-tolerant yeast responsible for lager beer fermentation. The largest share of the genetic diversity of *S. eubayanus* is located in South America, and strains of this species have not yet been found in Europe. One or more hybridization events between *S. eubayanus* and *S. cerevisiae* ale beer strains gave rise to *S. pastorianus,* the allopolyploid yeasts responsible for lager beer production worldwide. The identification of the missing progenitor of lager yeast opened new avenues for brewing yeast research. It allowed not only the selective breeding of new lager strains, but revealed also a wild yeast with interesting brewing abilities so that a beer solely fermented by *S. eubayanus* is currently on the market.

## Taxonomy

Kingdom *Fungi*, Phylum *Ascomycota*, Subphylum *Saccharomycotina*, Order *Saccharomycetales*, Family *Saccharomycetaceae*, genus *Saccharomyces*, species *Saccharomyces eubayanus*.

## Properties

*S. eubayanus*, together with *S. arboricola*, *S. kudriavzevii* and *S. uvarum*, forms the group of cold-tolerant species of *Saccharomyces,* i.e. species that are better adapted to growing at low temperatures (8–15 °C) and/or that have a maximum growth temperature in the range 33–35 °C, whereas the maximum growth temperatures for *S. cerevisiae* and *S. paradoxus* are 41–42 and 37–38 °C, respectively. Phylogenetically this species is basal in the genus, thus suggesting that the adaptations to higher temperatures seen in *S. cerevisiae* are derived traits.

## Genome

A 11.66 Mb *de novo* assembled genome sequence of the type strain (PYCC 6148^T^) was recently obtained [1], with a quality substantially higher than previously published genome sequences of this species [2, 3]. The genome of *S. eubayanus* is mostly syntenic with *S. cerevisiae*, with the exception of a few small inversions and two reciprocal translocations between chromosomes VIII and XV, and chromosomes II and IV [3]. *S. eubayanus* has a diploid genome wih a very low heterozygosity ratio of 0.0021 % [3]. Of the 5515 predicted protein-coding genes, a figure similar to current draft genomes of other *Saccharomyces* species, 4993 are unambiguous 1 : 1 : 1 orthologues among *S. cerevisiae, S. uvarum* and *S. eubayanus* [1]. The genes necessary for the utilization of maltose, collectively designated as *MAL* genes, are present in considerable numbers (14 genes spread across four chromosomes) and in sub-telomeric regions [1].

## Phylogeny and phylogeography

Together with *S. uvarum*, *S. eubayanus* occupies a basal position within the genus *Saccharomyces*. These two species also share their diversity hotspots in South America and an ecological association with *Nothofagus* spp. (Southern beech) [4, 5]. Although *S. eubayanus* has also been detected in other regions [5], the relatively low number of strains isolated and their lower genetic diversity suggests that South America is the primary radiation centre. In Patagonia, two 1 % divergent populations (Patagonia A and Patagonia B) have been detected. Members of population B have been also found in other regions, namely North America, Tibet and New Zealand, albeit infrequently. In Asia, another two more divergent populations (Sichuan and West China populations) were also detected.

## Key features and discoveries

The yeast that ferments lager-style beers represents a highly successful inter-species hybrid that was generated in the brewing environment and can therefore be viewed as a case of unintentional artificial selection. This yeast is presently classified in the exclusively domesticated species *S. pastorianus* (synonym *S. carlsbergensis*). This hybrid, that is thought to be intimately associated with the emergence of lager beer in Bavaria in the XVth century, has two progenitors: *S. cerevisiae* ale yeasts, the prototypical brewing yeasts, and *S. eubayanus* [2]. The identification of the non-*cerevisiae* progenitor of *S. pastorianus* remained contentious since the early 1980s, when the hybrid nature of lager yeasts become evident. Only three decades later, with the discovery of *S. eubayanus*, the origin of lager yeast became settled. Before the description of *S. eubayanus* in 2011, *S. bayanus*, a species described in 1895, was thought to represent the non-*cerevisiae* ancestor of *S. pastorianus* but it is now evident that *S. bayanus* is also a domestication-related hybrid with contributions from *S. cerevisiae, S. eubayanus* and *S. uvarum* [2]. Like *S. pastorianus*, *S. bayanus* is not found outside the brewing environment.

The identification of the missing progenitor of lager yeast opened new avenues for brewing yeast research. The brewing properties of *S. eubayanus* were compared to those of *S. pastorianus*, and it was observed that *S. eubayanus* was less sensitive to colder temperatures (10 °C) and unable to use maltotriose. Being well adapted to the relatively low temperatures of Patagonian *Nothofagus* forests, *S. eubayanus* performed poorly at 22 °C when compared to *S. pastorianus. S. eubayanus* was also used to generate *de novo* lager hybrids in crosses with ale strains [3]. The hybrids inherited relevant brewing properties from both parents and showed apparent hybrid vigor, fermenting faster and producing more ethanol than the parents. The discovery of *S. eubayanus* opened not only new opportunities for industrial utilization through the selective breeding of new lager strains, but revealed also a wild yeast with interesting brewing abilities so that a beer solely fermented by *S. eubayanus* is currently in the market.

## Open questions

- What is the global distribution of *S. eubayanus* and what are its dispersion routes?
- What is (are) the ecological niche(s) of *S. eubayanus* in regions outside Patagonia?
- Does *S. eubayanus* exist in Central Europe? This is the region where lager beer was first produced being therefore reasonable to assume that the hybrid of *S. eubayanus* and *S. cerevisiae* was formed there.
- Can laboratory-generated hybrids of *S. eubayanus × S. cerevisiae* outperform commercial *S. pastorianus* lager strains for beer production?
